# Effect of Umbilical Cord Entanglement and Position on Pregnancy Outcomes

**DOI:** 10.1155/2015/342065

**Published:** 2015-07-09

**Authors:** Natsuko Kobayashi, Shigeru Aoki, Mari S. Oba, Tsuneo Takahashi, Fumiki Hirahara

**Affiliations:** ^1^Perinatal Center for Maternity and Neonates, Yokohama City University Medical Center, 4-57 Urafunecyou, Minami-ku, Yokohama, Kanagawa 232-0024, Japan; ^2^Department of Biostatistics and Epidemiology, Yokohama City University Graduate School of Medicine and University Medical Center, Yokohama, Japan; ^3^Department of Obstetrics and Gynecology, Yokohama City University Hospital, Yokohama, Japan

## Abstract

*Introduction.* To investigate the effect of complex umbilical cord entanglement primarily around the trunk on pregnancy outcomes.* Methods*. We studied 6307 pregnant women with singleton pregnancies who underwent vaginal delivery of an infant at ≥37 weeks of gestation. Cases were classified into no cord, nuchal cord, and body cord groups and defined as cases without umbilical cord entanglement, one or more loops of the umbilical cord around the neck only, and umbilical cord around the trunk only, respectively. Pregnancy outcomes were compared among these three groups.* Results*. The no cord, nuchal cord, and body cord group included 4733, 1451, and 123 pregnancies, respectively. Although delivery mode was not significantly different among the three groups, 1-minute Apgar scores <7 and umbilical artery (UA) pH <7.10 were significantly more common in the umbilical cord entanglement groups than in the no cord group. In particular, the frequency of 5-minute Apgar scores <7 was significantly higher (*P* = 0.004), whereas that of UA pH <7.10 tended to be higher (*P* = 0.057) in the body cord group than in the nuchal cord group.* Conclusion*. Compared to nuchal cord, umbilical cord entanglement around the trunk was associated with a higher risk of low Apgar scores and low UA pH.

## 1. Introduction

Umbilical cord entanglement is the most common pathological condition among umbilical cord abnormalities [1], with an incidence ranging from 14.7% to 33.7% of all deliveries [1–3]. Umbilical cord entanglement reportedly increases the risk of prolonged labor and nonreassuring fetal status due to umbilical cord compression [1, 3–12], while some reports indicate that the risk of cesarean section or forced delivery is not increased [1, 5, 7, 13–16]. Therefore, consensus has not been reached. In addition, to the best of our knowledge, the majority of reports regarding umbilical cord entanglement concern nuchal cord entanglement, with no reported case concerning any other type of umbilical cord entanglement. Therefore, this study aimed to investigate the effect of complex umbilical cord entanglement primarily around the trunk on pregnancy outcome.

## 2. Materials and Methods

Data were retrospectively analyzed using the medical records of 8636 women with singleton pregnancies who had undergone attempted vaginal delivery at ≥37 gestational weeks between January 2004 and December 2013 at Yokohama City University Medical Center. Women with a serious complication, such as hypertension or diabetes, who delivered a newborn with congenital anomalies or with fetal malpresentation, were excluded. Consequently, 6307 of the 8636 women were included in this study. This study has been approved by the ethics committee of the Yokohama City University Medical Center. The presence or absence of umbilical cord entanglement was determined at the level of the umbilicus during delivery. The no cord group included cases without umbilical cord entanglement. The nuchal cord group included cases with at least one loop of the umbilical cord around the neck only. The body cord group included cases with the umbilical cord wrapped around the trunk, excluding the neck. Cases with umbilical cord entanglement around multiple parts, such as entanglement around both the neck and trunk or around both the neck and upper/lower limbs, were excluded. Pregnancy outcomes were compared among the 3 groups: no cord, nuchal cord, and body cord groups.

The following maternal characteristics were collected: maternal age at delivery, parity, and gestational age at delivery. The main outcome measures were delivery mode, birth weight, birth height, 1-minute Apgar scores <7, 5-minute Apgar scores <7, umbilical artery (UA) pH <7.1, and an excessively long umbilical cord. An excessively long umbilical cord was defined as an umbilical cord measuring ≥70 cm in length. Data are expressed as mean ± standard deviation or frequency (percentage). The IBM SPSS Statistics version 19 program was used for statistical analyses. Categorical variables were compared using *χ* ^2^ tests. Analysis of variance and* t*-tests were used to compare continuous variables. Statistical tests were considered significant at a *P* value < 0.05.

## 3. Results

The no cord group included 4733 pregnancies, the nuchal cord group included 1451 pregnancies, and the body cord group included 123 pregnancies.  Table 1 shows the maternal characteristics. No significant difference was observed among the groups in maternal age at delivery, parity, or gestational age.

Table 2 shows the main outcome measures for pregnancy outcomes among the 3 groups. No significant difference in delivery mode was observed among the groups. Moreover, the groups with umbilical cord entanglement, which were the nuchal cord and body cord groups, had significantly longer umbilical cords, compared with the no cord group. In particular, the nuchal cord group had the longest umbilical cord and included significantly more cases of excessively long umbilical cord. Significant differences in the frequencies of 1-minute and 5-minute Apgar scores <7 and <7, respectively, and UA pH <7.1 were observed between the 3 groups, with higher frequencies observed in the body cord group than in the other 2 groups. Significant differences were observed in neonatal birth weight between the no cord group and umbilical cord entanglement groups (*P* = 0.004), and birth weight was lower in the nuchal cord and body cord groups than in the no cord group. There were no significant differences in neonatal birth height among the 3 groups.

## 4. Discussion

Although delivery mode was not significantly different among the 3 groups, the frequencies of 1-minute Apgar scores <7 and UA pH <7.10 were significantly higher in the groups with umbilical cord entanglement than in the no cord group. In particular, the frequency of 5-minute Apgar scores <7 was significantly higher (*P* = 0.004) and frequency of UA pH <7.10 tended to be higher (*P* = 0.057) in the body cord group than in the nuchal cord group.

In this study, the presence or absence of umbilical cord entanglement did not affect the delivery mode. This finding is similar to that of the majority of previous studies, in which there were no differences in cesarean section rates based on the presence or absence of umbilical cord entanglement [1, 5, 6, 13–16]. Meanwhile, Larson et al. [4] reported that the instrumental delivery rate was higher in cases with multiple umbilical cord entanglement, but cesarean section rates were not significantly different. Moreover, Bernad et al. [7] reported that umbilical cord entanglement might be a cause of intrauterine fetal death even though there was no difference in forced delivery rates based on cord entanglement. The authors recommended that rigorous management with fetal heart rate monitoring should be conducted during delivery when ultrasonography clearly reveals umbilical cord entanglement and cesarean section should be considered when nonreassuring fetal status is detected.

In the groups with umbilical cord entanglement, the frequencies of 1-minute Apgar scores <7 and UA pH <7.10 were higher than in the no cord group. Assimakopoulos et al. [6] reported that cases with umbilical cord entanglement more frequently had low Apgar scores and low UA pH as have many other studies for either low Apgar scores or low UA pH [1, 4, 6, 8, 10, 12]. The results of the present study also support the findings of these studies and confirm that the presence or absence of umbilical cord entanglement affects neonatal conditions at delivery.

The frequency of 5-minute Apgar scores <7 was significantly higher in the body cord group compared with the nuchal cord group in the present study, and the frequency of UA pH <7.10 also tended to be higher. To our knowledge, the majority of studies regarding umbilical cord entanglement concern nuchal cord entanglement, and no previous study has investigated umbilical cord entanglement around the trunk. The lower Apgar scores and UA pH in the body cord group than in the nuchal cord group might be explained by a greater likelihood to suffer umbilical cord compression during uterine contraction in fetuses with umbilical cord entanglement around the trunk compared with nuchal cord entanglement, because a space between the head and trunk is not present in the former but is in the latter.

Neonatal birth weight was 34 g and 45 g lower in the nuchal cord and body cord groups, respectively, than in the no cord group. In a study of neonatal outcomes based on the presence or absence of umbilical cord entanglement in 57853 deliveries, Ogueh et al. [5] reported that the birth weight of fetuses with nuchal cord entanglement was 55 g lower than without nuchal cord entanglement. The authors suggested that chronic intermittent cord compression with hypoxia might lead to fetal growth restriction; alternatively, smaller fetuses have more space to move around in the uterus and are consequently more likely to have umbilical cord entanglement. Meanwhile, Sheiner et al. [1] reported in a similar study which included 166318 deliveries that the birth weight of fetuses with nuchal cord entanglement tended to be higher. Although the results of the present study support those reported by Ogueh et al. [5], further studies are needed to establish firm conclusions regarding the relationship between umbilical cord entanglement and fetal growth.

The present study has several limitations. First, it was conducted with a small sample in a single institution. Second, the effects of nuchal cord entanglement were not evaluated based on the number of loops. Moreover, cases with multiple umbilical cord entanglements involving multiple parts of the body, such as entanglement around both the neck and upper/lower limbs, were excluded.

In conclusion, umbilical cord entanglement is associated with an increased risk of low Apgar scores and low UA pH. The present study suggests that fetuses with complex umbilical cord entanglement primarily around the trunk, but not the neck, are strongly affected by umbilical cord compression during delivery. However, delivery modes were not affected by any type of umbilical cord entanglement, which supports the findings of previous studies. Umbilical cord entanglement is a common pathological condition encountered in daily clinical practice. Although it might affect neonatal conditions during delivery, vaginal delivery can be safely performed in many cases, and undue concern should not be passed on to the mothers, even when ultrasonography reveals the presence of umbilical cord entanglement before delivery.

## Figures and Tables

**Table 1:** Maternal characteristics, compared between the 3 groups.

	No nuchal cord	Nuchal neck cords	Nuchal body cords	*P* value
	(*n* = 4733)	(*n* = 1451)	(*n* = 123)	
Maternal age	31.7 ± 5.1	32.0 ± 5.2	32.4 ± 5.3	0.548
Parity				
Primiparous	2356 (49.8%)	773 (53.3%)	64 (52%)	0.272
Multiparous	2377 (50.2%)	678 (46.7%)	59 (48%)	0.882
Gestational age (weeks)	39.6 ± 1.1	39.7 ± 1.1	39.4 ± 1.0	0.064

**Table 2:** Comparison of pregnancy outcomes between the 3 groups.

	No nuchal cord	Nuchal neck cords	Nuchal body cords	*P* value
	(*n* = 4733)	(*n* = 1451)	(*n* = 123)	
Mode of delivery				
Spontaneous vaginal delivery	4110 (86.8%)	1248 (86.0%)	105 (85.4%)	
Instrumental delivery	298 (6.3%)	89 (6.1%)	7 (5.7%)	0.868
Cesarean delivery	325 (6.9%)	114 (7.9%)	11 (8.9%)	*0.605 *
Excessively long umbilical cord	54 (1.1%)	403 (27.8%)	25 (20.3%)	<0.01
Umbilical cord length (cm)	53.7 ± 8.6	64.1 ± 11.2	61.0 ± 10.8	
Apgar score at 1 min <7	80 (1.7%)	41 (2.8%)	6 (4.9%)	0.002
Apgar score at 5 min <7	9 (0.2%)	6 (0.4%)	3 (2.4%)	*<0.01 *
UApH <7.10	58 (1.2%)	24 (1.6%)	5 (4.1%)	
	7.30 ± 0.070	7.29 ± 0.071	7.28 ± 0.075	*0.024 *
Birth weight (g)	3053 ± 366	3019 ± 369	3008 ± 361	*0.004 *
Birth height (cm)	48.9 ± 1.9	48.8 ± 2.1	48.8 ± 2.0	*0.261 *

UApH: umbilical artery pH.
